# Strongyloidiasis in Ethiopia: systematic review on risk factors, diagnosis, prevalence and clinical outcomes

**DOI:** 10.1186/s40249-019-0555-3

**Published:** 2019-06-14

**Authors:** Yitagele Terefe, Kirstin Ross, Harriet Whiley

**Affiliations:** 1 College of Veterinary Medicine, Haramaya University, P.O. Box 138, Dire Dawa, Ethiopia; 2 Foodborne Pathogen Diagnosis Research Laboratory, Haramaya University, P.O.Box 138, Dire Dawa, Ethiopia; 3 Environmental Health, College of Science and Engineering, Flinders University, GPO Box 2100, Adelaide, South Australia 5001 Australia

**Keywords:** Neglected tropical disease, Soil transmitted helminth, Public health, *Strongyloides stercoralis*, AIDS, HIV, Anemia

## Abstract

**Background:**

Strongyloidiasis is a gastrointestinal infection caused by the parasitic nematode *Strongyloides stercoralis.* It is estimated to infect up to 370 million people globally and is predominately found in tropical and subtropical areas of socioeconomic disadvantage.

**Main body:**

This systematic literature review identified studies published in the last ten years on the risk factors, diagnosis, prevalence and/or clinical outcomes of strongyloidiasis in Ethiopia. The prevalence of *S. stercoralis* ranged from 0.2 to 11.1% in adults, 0.3% to 20.7% in children, 1.5% to 17.3% in HIV positive adults and 5% in HIV positive children. The identified studies primarily used microscopy based techniques that potentially underestimated the prevalence four fold compared with serology and PCR. Strongyloidiasis in children presents a particularly significant issue in Ethiopia as children often presented with anemia, which is associated with impaired mental and cognitive development. The most significant risk factor for strongyloidiasis was HIV status and although other risk factors were identified for helminth infections, none were statistically significant for *S. stercoralis* specifically. Several studies detected *S*. *stercoralis* in dogs and non-biting cyclorrhaphan flies. However, future research is needed to explore the role of these reservoirs in disease transmission.

**Conclusions:**

This review demonstrated that strongyloidiasis is an overlooked and neglected disease in Ethiopia. There is a need for a systematic approach using a combination of molecular and serology based diagnostic methods to ascertain the true incidence and burden of strongyloidiasis in Ethiopia. Further research is also needed to break the cycle of transmission by identifying environmental reservoirs, risk factors and exploring the potential for zoonotic transfer.

**Electronic supplementary material:**

The online version of this article (10.1186/s40249-019-0555-3) contains supplementary material, which is available to authorized users.

## Multilingual abstracts

Please see Additional file 1 for translations of the abstract into the five official working languages of the United Nations.

## Background

Strongyloidiasis is caused by infection with the parasitic nematode worm, *Strongyloides stercoralis, S. fuelleborni* or *S. fuelleborni kelli* [1, 2]. Symptoms of infection range from asymptotic to non-specific gastrointestinal complaints [2, 3] and distinctive form of cutaneous larva migrans, larva currens [4]. The infection can remain undetected and undiagnosed for many decades [5, 6]. However, when an infected person undergoes steroidal or immunosuppressive treatment, the worm infection undergoes hyperinfection, leading to enormous numbers of the parasite, which can then disseminate and move to other organs, a condition that is almost always fatal [7].

Strongyloidiasis has been assumed to be a disease associated with tropical or subtropical regions, and is recognised as a neglected tropical disease. However, it has been argued that strongyloidiasis should be described as a disease of disadvantage, as it is primarily a disease from developing countries and from poorer areas within developed countries [8]. Estimates of infection rates globally range from 50–100 million [3, 4] to more than 300 million [9]. The lower estimate is likely to be an underestimate as the disease is often undiagnosed, either because it is not looked for [3, 10] or because of difficulties with diagnosis [2, 11].

Ethiopia’s economy is one of the fastest growing in the world [12] although it is also one of the poorest countries in Africa [13]. The Ethiopian health care sector is currently underfunded by both global and regional standards [14] and housing and other environmental health hardware components in many areas is substandard in both rural and urban areas [15, 16]. The lack of health care and poor environmental health conditions combine to make the country susceptible to parasitic infections, including strongyloidiasis. Here we evaluate the prevalence, risk factors and clinical outcomes for strongyloidiasis in Ethiopia reported in the literature using a systematic approach.

## Main text

### Materials and methods

#### Search strategy

**Fig. 1:**
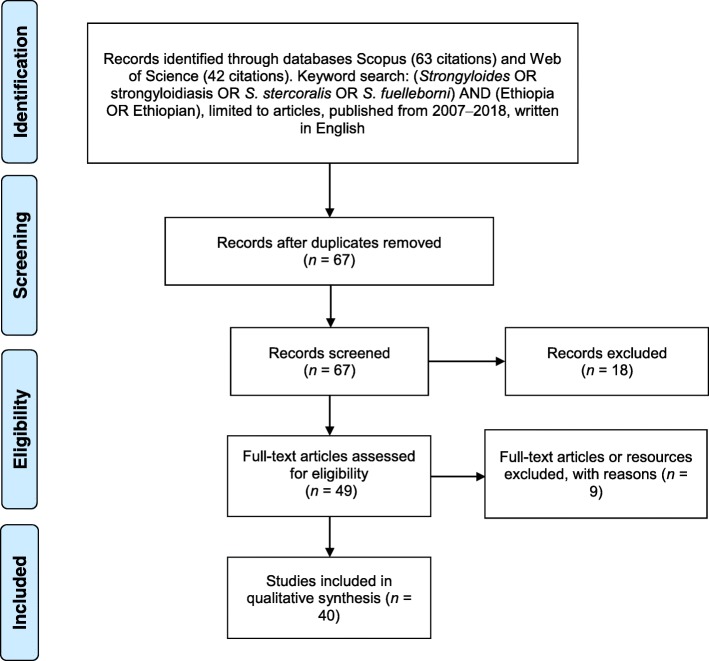
Overview of search methods and articles inclusion and exclusion criteria

The databases Scopus (*n* = 46) and Web of Science (*n* = 44) were searched for articles written in English over the last ten years containing the keywords *Strongyloides* OR strongyloidiasis OR *S. stercoralis* OR *S. fuelleborni* AND Ethiopia OR Ethiopian. Figure 1 presents the systematic approach to article inclusion or exclusion. Articles were screened by reading titles and abstracts and initially excluded if they did not refer to specifically to *S. stercoralis* or if they were review articles. Article were further screened by reading abstracts and full articles and excluded if they did not investigate the prevalence of strongyloidiasis, or describe clinical presentations or potential environmental sources. As the aim of this systematic review was to provide an overview of the state of knowledge relating to strongyloidiasis in Ethiopia, all articles which met the inclusion criteria were included regardless of any flaw in study design. Limitations or bias identified are discussed in the discussion section.

## Results

### Prevalence

A total of 40 publications published in the last 10 years were identified that investigated the prevalence of strongyloidiasis, described clinical presentations or potential environmental sources. Table 1 presents the 27 publications that examined the prevalence of strongyloidiasis in different Ethiopian populations. In HIV negative adults the prevalence of strongyloidiasis ranged from 0.2% to 11.1%. Combining the results from the 16 studies investigating HIV negative adults demonstrated that the overall prevalence was 0.5% (195 positive individuals out of 36 549 tested). However, this value is skewed by the large samples size (32 191 people) and low incidence observed in the study by Ramos et al [33]. This study did not investigate the general population but rather patients from Gambo Rural Hospital presenting with diarrhea. The omission of this study increased the overall prevalence in healthy adults to 2.5%. It is also important to note that in all of these studies investigating HIV negative adults, *Strongyloide*s was detected using microscopy based stool examination techniques, which is likely to significantly underestimate the prevalence due to false negative results.

**Table 1:** Studies investigating the prevalence of strongyloidiasis in different populations across Ethiopia

No.	Prevalence	Population	Detection method		Location	Reference
			Parasitological	Immunological/Molecular		
1	1.9% total 2.8% in males; 0.9% in females 3.2% in farmers	427 patients 15 years and above who were requested for stool examination in Adwa Health Center from March 2013 to December 2015.	Stool examination (wet mount and Kato-Katz microscopy techniques)	NA	Adwa, Northern Ethiopia	[17, 18]
2	4.8% (stool) 16.5% (serology)	315 Ethiopian children adopted in Belgium 2008–2014	Stool examination	Serology (IgG antibodies)	Unknown	[19]
3	12.3% total 17.3% HIV positive 3.2% in HIV negative	351 subjects (226 HIV positive and 125 HIV negative)	Stool examination (agar plate culture and Baermann's method)	NA	Addis Ababa, Central Ethiopia	[20]
4	0.7%	408 schoolchildren	Stool examination (formalin-ether concentration microscopy technique)	NA	Zegie Peninsula, North Western Ethiopia	[21]
5	3.6%	223 HIV/AIDS clients at the University of Gondar Hospital, Northwest Ethiopia.	Stool examination (wet mount and formalin-ether concentration and Ziehl-Neelson staining microscopy techniques)	NA	Gondar Hospital, North Western Ethiopia	[22]
6	1.5%	323 HIV infected participants on HAART	Stool examination (formalin-ether concentration microscopy technique)	NA	Butajira Hospital, Southern Ethiopia	[23]
7	0.94	213 pulmonary TB patients	Stool examination (formalin-ether concentration microscopy technique)	NA	Arba Minch, South Western Ethiopia	[17]
8	1.6 %	384 pregnant women	Stool examination (formalin-ether concentration microscopy technique)	NA	Felege Hiwot Referral Hospital, Northern Ethiopia	[24]
9	20.7% (3.5% by stool, 12.1% by Baermann and 13.4% by PCR)	396 primary school children aged 13–14	Stool examination (stool examination (formalin-ether concentration and Baermann microscopy technique)	PCR using *S. stercoralis-*specific primers targeting the 18S ribosomal subunit, as described by Verweij et al. [25]	Rural highland of North Western Ethiopia	[26, 27]
10	20.6%	605 Ethiopian refugee children in USA 2006–2012	NA	Serology (antibody information not provided)	Unknown	[28]
11	1.9%	277 children under 5 suspect with IP at University of Gondar hospital	Stool examination (wet mount and formalin-ether concentration microscopy techniques)	NA	Gondar, North Western Ethiopia	[29]
12	1.1%	Anbesame Health Center from March to June 2015. A structured questionnaire was used to collect data from 464 study participants selected consecutively	Stool examination (formalin-ether concentration microscopy technique)	NA	Dera district, Gondar, North Western Ethiopia	[30]
13	2.5% in HARRT initiated and 6.9% in HARRT Naïve	180 pediatric HIV/AIDS patients	Stool examination (formalin-ether concentration microscopy technique)	NA	Addis Ababa, Central Ethiopia	[31]
14	5.6% in prisoner and 1.7 in tobacco farm	236 Prison inmates and tobacco farm workers	Stool examination (formalin-ether concentration microscopy technique)	NA	Shewa Robit, Central Ethiopia	[32]
15	0.2% in female and 0.3% in male	32,191 patients who presented with diarrhea 2007–2012 in Gambo Rural Hospital	Stool examination (direct microscopy using saline smear mount and Lugol’s iodine staining)	NA	Gambo, West Arsi Province, Southern Ethiopia	[33]
16	3.47% total 5.1% in rural 2% in urban	778 primary school children age 7–14	Stool examination (Kato-Katz and formalin-ether concentration microscopy technique)	NA	Bahir Dar, North Western Ethiopia	[34]
17	5.9% total 11.1% in low land 0% in highland	464 members of a lowland communities (Lante and Kolla Shelle) 403 members of highland communities (Dorze and Geressie)	Stool examination (wet mount and formalin-ether concentration microscopy technique)	NA	Gamo, Southern Ethiopia	[35]
18	0.5%	200 food handlers working in University of Gondar student cafeterias.	Stool examination (formalin-ether concentration, sedimentation or smear mount in saline microscopy techniques)	NA	Gondar University, North Western Ethiopia	[36]
19	3.10%	384 consecutive diarrheal patients who visited Gondar Teaching Hospital	Stool examination (direct microscopy)	NA	Gondar, North Western Ethiopia	[37]
20	0.69%	288 under-five children	Stool examination (Kato-Katz and formalin-ether concentration microscopy technique)	NA	Shesha Kebkele, Wondo Genet, Southern Ethiopia	[38]
21	0.30%	386 Primary school children 7–18 years old	Stool examination (formalin-ether concentration microscopy technique)	NA	Adwa, Northern Ethiopia	[39]
22	2.86%	300 food handlers	Stool examination (formalin–ether concentration microscopy technique)	NA	Bahir Dar, North Western Ethiopia	[40]
23	12.0% in HIV positive 2.1% in HIV negative	384 individual (192 HIV positive and 192 HIV negative) Yirgalem Hospital	Stool examination (saline smear mount, the formalin-ether concentration or water emergence microscopy techniques)	NA	Yirgalem Hospital, Southern Ethiopia	[41]
24	1%	401 healthy individuals	Stool examination (formalin-ether concentration microscopy technique)	NA	Kara and Kwego tribes, Lower Omo River Valley, South Western Ethiopia	[42]
25	7.4% total 12.6% in HIV positive 0.6% in HIV negative	378 consecutive participants Hawassa Teaching and Referral Hospital (214 HIV positive and 164 HIV negative)	Stool examination (formalin-ether concentration microscopy technique)	NA	Hawassa Teaching and Referral Hospital, Central Ethiopia	[43]
26	5.5%	127 food handlers	Stool examination (formalin-ether concentration microscopy technique)	NA	Gondar, North Western Ethiopia	[44]
27	11.5% HIV/AIDS positive 1.8% HIV 0% in HIV negative	160 subjects from Jimma Hospital, Mother Theresa Missionary Charity Centre, Medan Acts Projects, Mekdim HIV Positive Persons and AIDS Orphans National Association. (52 HIV/AIDS positive, 57 HIV positive health carrier and 51 HIV negative individuals)	Stool examination (formalin-ether concentration microscopy technique)	NA	Jimma, South Western Ethiopia	[45]

*AIDS* Acquired immunodeficiency syndrome, *HAART* Highly active antiretroviral therapy, *HIV* Human immunodeficiency virus, *HTLV-1* Human T-cell lymphotropic virus type 1, *IP* Intestinal parasite, *NA* Not applicable, *PCR* Polymerase chain reaction, *TB* Tuberculosis

In HIV negative children the prevalence of strongyloidiasis ranged from 0.3% to 20.7% and the overall combined prevalence from 8 studies was 8.6% (298 positive out of 3453 children). However, there were significant variations in incidence depending on the diagnostic technique used. The overall prevalence in children using microscopy based stool examination techniques was 4% (from seven studies, two of which concurrently used PCR or serology) (Table 1); whereas the overall prevalence using PCR detection from stools or serology was 17.5% (three studies, two of which concurrently used microscopy). The two studies which tested using more than one detection method found 5% prevalence using microscopy compared with 17% using serology [19], and 4% using microscopy compared to 13% using PCR [26]. This suggests that microscopy based techniques could potentially underestimate prevalence by up to four fold compared with serology or PCR. This is consistent with the findings of Van Kesteren and Wojciechowski [19] that concurrently tested faeces using microscopy examination of stools and serology and found positives in 4.8% and 16.5% of patients respectively. Another study by Amor et al. reported detection of 3.5% by stool, 12.1% by Baermann and 13.4% by PCR [26]. The most common diagnostic technique by far was stool examination using the formalin-ether concentration technique, followed by the wet mount, Kato-Katz and Baermann with only one or two studies each using saline smear mount, sedimentation, agar, PCR or serology (Table 1).

The highest prevalence seen using microscopy based stool examination was in HIV positive adults. The prevalence ranged from 1.5% to 17.3% and the overall combined prevalence was 11%. There was only one study of pediatric HIV patients which found the incidence to be 5% (2.5% in highly active antiretroviral therapy [HARRT] initiated and 6.9% in HARRT naïve patients) [31].

### Risk factors

The most significant risk factor for strongyloidiasis identified was HIV status (Table 1). Four studies compared the prevalence of strongyloidiasis in HIV positive and HIV negative individuals and the incidence was 5–20 times higher in HIV positive individuals compared with HIV negative [20, 41, 43, 45].

Two studies reported that the prevalence of strongyloidiasis in otherwise healthy individuals was slightly higher in males compared to females [17, 18, 33] and that higher incidence was seen in rural areas compared with urban areas [34] and in farmers [17, 18]. Three studies investigated food handlers; however, the overall prevalence was 2.7%, which is not significantly higher than the general population [36, 40, 44].

Several studies identified additional risk factors associated with the prevalence of intestinal parasites. Not wearing shoes, not washing hands [21, 34], not trimming fingernails, or having dirt under the nails [34] were statistically significantly associated (*P* < 0.05) with intestinal helminths (including *Strongyloides*). Eating unwashed/raw fruit, open field defecation, and living in a rural area was statistically significantly associated (*P* < 0.05) with intestinal parasites (including *Strongyloides* and other parasites) [31]. However, there were no statistically significant risk factors associated with strongyloidiasis specifically. This could be a result of the small sample sizes due to the lower prevalence of strongyloidiasis. It also highlights the need for more systematic approaches to epidemiological studies investigating the prevalence and risk factors for strongyloidiasis.

### Clinical manifestations

Table 2 presents studies describing clinical manifestations of strongyloidiasis in Ethiopian individuals. The most common clinical presentation was anemia, which was observed in immunocompetent adults and children and HIV positive children. Immunocompromised individuals presented with a range of symptoms and clinical manifestations including eosinophilia, fever, vomiting, hematemesis, diarrhea, abdominal pain, bacteremia, sepsis, cough, respiratory distress, chronic obstructive pulmonary disease, hypoxemia, diffuse alveolar hemorrhage and meningitis (Table 2). Two studies reported cases of hyperinfection, one of which was fatal [51, 52]. Another study by Nadir & Zimhony [50] reported eight cases of strongyloidiasis in AIDS patients, seven of which were fatal. The diagnosis of strongyloidiasis in these cases was complicated by negative serology results but diagnosis was confirmed through PCR and microscopy. Negative serology results were also observed in two reported cases of strongyloidiasis in immunocompetent children and diagnosis was achieved through stool examination [47, 48, 50].

**Table 2:** Studies illustrating clinical manifestations and outcomes of strongyloidiasis in Ethiopians

Population	Clinical manifestations	Detection method	Reference
427 patients 15 years and above who were requested for stool examination in Adwa Health Center during the study period.	Study participants infected with *S. stercoralis* were more likely to develop anemia than the non-infected participants; a*OR* (adjusted odds ratio) = 5.3, 95% *CI* (1.01–27.4);	Stool examination (wet mount and Kato-Katz microscopy techniques)	[17, 18]
14-year-old Ethiopian girl adopted in Italy	Severe anemia (hemoglobin 4.9 g/dL) and a lung nodule	Stool examination (qPCR positive (primer information not provided) but negative for formalin-ether microscopy) Serology positive (IgG antibodies)	[46]
13 months old Ethiopian adoptee in Canada	Eosinophilia and bloating	Stool examination and serology (serology was negative – antibody information not provided)	[47]
21 month Ethiopian adoptee in Spain	No eosinophilia, loose and pasty stool, anemia	Stool examination (charcoal culture) and serology (serology negative - antibody information not provided)	[48]
4 immunocompromised Ethiopian immigrants in Israel	Meningitis	qPCR of stool and Cerebrospinal fluid (qPCR using *S. stercoralis*-specific primers targeting the 18S ribosomal subunit, as described by Verweij et al. (2009))	[49]
8 AIDS patients with severe strongyloidiasis 7/8 cases were fatal	1. Fever, vomiting and hematemesis, abdominal pain, *E. coli* bacteremia and respiratory distress 2. Recurrent admissions due to fever, abdominal pain, vomiting, respiratory distress and cough 3. Vomiting and diarrhea 4. *E. coli* bacteremia of unknown origin 5. Vomiting and diarrhea 6. Postpartum sepsis, *K. pneumonia* bacteremia and & respiratory failure 7. *E. coli* bacteremia followed by ESBL pos*. E. coli* meningitis 8. Abdominal pain and hematemesis, followed by sepsis-like syndrome and respiratory failure	Serology negative (or not determined) in all cases (antibody information not provided) Duodenal biopsy, intestinal biopsy, stool O&P, duodenal aspirate, gastric aspirate, CSF sputum	[50]
50-year-old Ethiopian immigrant in USA	Recurrent GIT bleeding; eosinophilia, central obesity, severe proximal muscle wasting and weakness - hyperinfection	Histopathological examination of gGIT biopsies	[51]
180 pediatric HIV/AIDS patients	Anemia	Stool examination (formalin-ether concentration microscopy technique)	[31]
36-year-old HIV patient	Diffuse alveolar haemorrhage and severe hypoxemia - hyperinfection	Bronchoalveolar lavage cytology	[52]
31-year-old male Ethiopian immigrant in Canada	Mild eosinophilia and diarrhea	Serology (antibody information not provided)	[53]
Immunocompromised Ethiopian immigrant in Israel	Intermittent eosinophilia, bronchial spasm and chronic obstructive pulmonary disease	Duodenal biopsy and duodenal aspirates	[54]
378 consecutive participants Hawassa Teaching and Referral Hospital (214 HIV positive and 164 HIV negative)	Diarrhea and lower CD4 count	Stool examination (formalin–ether concentration microscopy technique)	[43]

*AIDS* Acquired immunodeficiency syndrome, *CSF* Cerebrospinal fluid, *GIT* Gastrointestinal tract, *HIV* Human immunodeficiency virus, *O&P* Ova and parasite examination, *qPCR* Quantitative polymerase chain reaction

### Environmental sources

There were four studies that detected *S. stercoralis* in potential environmental sources. Two studies detected *S. stercoralis* in dog faeces [55, 56] and two in non-biting cyclorrhaphan flies [57, 58], all of which used microscopy based stool examination techniques. A study conducted in Hawassa examined 448 dogs and found 31% were positive for *S. stercoralis* [56]. Another examined 384 pet dogs and 46 stray dogs in Bahir Dar and found 30% and 46% positive respectively [55]. A study in Woreta examined 6530 non-biting cyclorrhaphan flies and found the overall presence of *S. stercoralis* to be 1.7%. This included 12% of *Chrysomya rufifacies*, 16% *Musca sorbens* and 34% *Lucilia cuprina.* However, *Musca domestica, Calliphora vicina, Chrysomya bezziana* and *Wohlfahrtia magnifica* were all negative [58]. These findings support a previous study conducted in Addis Ababa which examined 9550 non-biting cyclorrhaphan flies and found that 0.1–0.2% of *C. rufifacies* and 0.6% of *M. sorbens* were positive for *S. stercoralis,* whereas *L. cuprina, M. domestica, C. vicina, Sarcophaga* spp., and *Wohlfahrtia* spp. were all negative. Interestingly, *S. stercoralis* positive flies were only found in defecating areas or garbage and were not found near the butchery or market [57].

## Discussion

This systematic review revealed that the prevalence of *S. stercoralis* in HIV negative Ethiopians ranged from 0.2 to 11.1% in adults and 0.3% to 20.7% in children and the calculated overall prevalence was 0.5–2.5% and 8.6% in adults and children respectively. This is comparable with other studies from Sierra Leone, Côte d’Ivoire and Sudan, which found the prevalence of *S. stercoralis* to be less than 5% [59–61]. However, it is lower compared with other studies from Angola, Nigeria and Ghana, which found the incidence of strongyloidiasis to be above 15% [25, 62, 63]. The differences in prevalence observed in different studies may be due to different laboratory techniques, study populations, geographical factors, or economic status. In studies exploring the prevalence in children a common clinical presentation identified was anemia (Table 1). This suggests that undiagnosed strongyloidiasis in children may have significant long term consequences as iron deficiency and anemia in early childhood is associated with impaired cognition and learning ability [64]. This presents an issue of significant public health, economic and social concern for Ethiopia as infection in children can adversely affect physical, mental, education and overall societal development [65].

In this review, HIV positive individuals represented the highest risk group for *S. stercoralis* infection in Ethiopia. This finding supports several previous studies conducted across the globe [66, 67]. It is interesting to note that the increased risk of strongyloidiasis in HIV positive individuals is not associated with an increase incidence of disseminated or hyperinfection [68]. Only one case of hyperinfection in a 36-year-old HIV positive individual was identified in this review [52]. However, a case report of strongyloidiasis in eight AIDS patients illustrated the severity of this disease in immunocompromised individuals with seven out of eight cases resulting in fatalities [50]. A complication of strongyloidiasis in HIV positive and immunocompromised individuals is the observation that serology can be falsely negative [50, 69]. Previous global studies have also identified human T-cell lymphotropic virus type 1 Infection (HTLV-1) and alcoholism as risk factors for strongyloidiasis [70]; however, this was not seen in the Ethiopian studies. Although the absence of these risk factors could be attributed to a lack of a systematic approach to epidemiological surveillance of strongyloidiasis in Ethiopia.

There was a significant discrepancy in the prevalence observed in Ethiopia based on the diagnostic methods used in a study. Comparisons between studies found that the microscopy based techniques could potentially underestimate the prevalence by four fold compared with serology or PCR. As the majority of studies identified in this review used microscopy based techniques the true incidence of strongyloidiasis could be much higher. This supports a recent study that suggests that *S. stercoralis* infection could be overlooked and neglected in Ethiopia [27]. Similarly, a review of the global perspectives of strongyloidiasis indicated that the disease was currently underestimated in many countries, but despite this underestimation, prevalence is steadily increasing [71].

In Ethiopia, a higher prevalence of *S. stercoralis* was recorded in farmers and in rural populations. These findings are supported by studies conducted in Cambodia and China which identified rural populations to be at greater risk for strongyloidiasis [72, 73]. This could be due to greater environmental contamination by the larvae of *S. stercoralis* and conditions supporting their survival. However, there is a need for environmental sampling to confirm this assumption and to identify the main environmental reservoirs. Global studies have also indicated that tropical and subtropical settings in areas of economic disadvantage provide ideal conditions for transmission dynamics of *S. stercoralis* [71, 74]. This highlights the needs for research identifying the best practice in managing these environmental systems to break the life cycle and transmission dynamics of this parasite.

There were several studies which demonstrated the incidence of strongyloidiasis in Ethiopian food handlers was not greater than that of the general public. This supports the findings of a study in Malaysia which detected *S. stercoralis* in water samples that were used in the production of different vegetables (pegaga, kesum and water spinach) suggesting that vegetables and herbs may be a source of strongyloidiasis in this region [75].

Two studies demonstrated the prevalence of *S. stercoralis* in Ethiopian dogs [55, 56]. This is noteworthy given recent studies that have demonstrated the potential zoonotic transmission of *S. stercoralis* from dogs to humans [76, 77]. This incidence of *S. stercoralis* in Ethiopian dogs should inform future strategies for the control of strongyloidiasis. This is particularly relevant to the argument that mass drug administration schemes to treat human should consider concomitant treatment of dogs [78]. The two studies that detected *S. stercoralis* in non-biting cyclorrhaphan flies [57, 58] provide further insight into the potential transmission and environmental reservoirs of this parasite. This association with flies found in defecating areas and garbage supports the argument that strongyloidiasis is associated with improper sanitation hardware [74] although there is a need for further research to explore survival and transmission from these sources. It is also significant when considering the argument made by Gove [79] almost thirty years ago that the most effective approach to controlling *Strongyloides* infection is to control the environmental sources.

The complied information on clinical manifestations suggests that strongyloidiasis commonly presents with anemia, diarrhea, vomiting and eosinophilia. It provides a body of evidence for control campaigns or clinical management schemes to critically consider high-risk groups, such as HIV positive children, who may benefit from routine testing and/or de-worming of helminth infection [80]. Currently there are no government programs specifically targeting the surveillance, treatment and intervention of strongyloidiasis in Ethiopia. However, the Federal Ministries of Health and Education in collaboration with different international organizations deliver deworming treatments to children to tackle soil-transmitted helminthiases (intestinal worms) and schistosomiasis (bilharzia). These treatments programs are being scaled up each year and have the target of reaching 26.1 million children annually (80% of all at-risk children) by 2020 [81]. However, to be able to evaluate the effectiveness of these deworming programs there is a need for a systematic approach using a combination of molecular and serology based diagnostic to ascertain the true incidence and burden of strongyloidiasis. Currently there is not enough information to inform medical practitioners and public health policy advisors. For example, there are limited reports from the Eastern and South Western parts of Ethiopia, although there are many hospital-based studies were reported from northern and southern part of the country which were mainly conducted by universities in the nearby areas. In addition to this, there are very few reports investigating the role of domestic animals and environmental reservoirs in the transmission of this disease.

## Conclusions

This review identified that strongyloidiasis is a potentially overlooked and neglected disease in Ethiopia. Currently, there is limited information on the epidemiological situation of strongyloidiasis across the country and the studies that are available are based on microscopy techniques that underestimate the true incidence. The incidence of strongyloidiasis in children presents a significant issue for Ethiopia as infection is associated with impaired mental and cognitive development, affecting the education and societal development of an individual. In order to break the cycle of disease transmission future research is needed to identify the environmental reservoirs and routes of exposure. This includes the potential zoonotic capacity of *Strongyloides* from dogs and cats and information about survival within different reservoirs.

## Additional file

Additional file 1: Multilingual abstracts in the five official working languages of the United Nations. (PDF 506 kb)

## Data Availability

Not applicable.

## Abbreviations

AIDS: Acquired immunodeficiency syndrome
CSF: Cerebrospinal fluid
GIT: Gastrointestinal tract
HAART: Highly active antiretroviral therapy
HIV: Human immunodeficiency virus
HTLV-1: Human T-cell lymphotropic virus type 1
IP: Intestinal parasite
O&P: Ova and parasite examination
PCR: Polymerase chain reaction
qPCR: Quantitative polymerase chain reaction
TB: Tuberculosis
