# Supplementary material for: Strongyloidiasis in Ethiopia: systematic review on risk factors, diagnosis, prevalence and clinical outcomes
Source: Infect Dis Poverty. 2019 Jun 14;8:53. doi: 10.1186/s40249-019-0555-3 (PMC6567471; doi:10.1186/s40249-019-0555-3)
Supplement: Supplementary file 1 — Multilingual abstracts in the five official working languages of the United Nations. (PDF 506 kb) [file 40249_2019_555_MOESM1_ESM.pdf]

Translation of the abstract into the five official working languages of the United Nations

الداء الخيطيفي إثيوبيا؛ مراجعة منهجية لعوامل الخطر، التشخيص، معدل الانتشار ونتائج الأبحاث الإكلينيكية.

يتاجلي ترفي، كرستين روس وهارييت وايلي.

نُبذة تمهيدية

خلفية علمية: هوعدوى معوية تسببها ديدان خيطية طفيلةالاسطوانيات البرازية. تشير التقديرات إلى إصابة ما يقارب من 370 مليون شخص حول العالم بهذه العدوى وقد وجدت غالبا في المناطق الأستوائية وشبه الاستوائية ذات الأوضاع الاجتماعية الاقتصادية السيئة.

الجزء الأساسي في البحث: تعرف هذه المراجعة الأدبية المنهجية بالدراسات التي نشرت خلال الأعوام العشرة الأخيرة حول عوامل الخطر، التشخيص، معدل الانتشار و/أو نتائج البحث الإكلينيكي لداء الخيطيات في إثيوبيا. تراوح معدل انتشار الأسطوانيات البرازية من 0.2 إلى 11.1% في البالغين ، من 0.3 إلى 20.7% في الأطفال، ومن 1.5 إلى 17.3% في البالغين من حاملي فيروس الHIV و 5% لدى الأطفال حاملي الفيروس.تعتمد الدراسات المبينة بشكل أساسي على التقنيات المجهرية والتي من المحتمل أنها تقلل معدل الإنتشار أربعة أضعاف عند مقارنته بالأمصال والPCR. يعد الداء الخيطي عند الأطفال مشكلة مهمة خاصة في إثيوبيا حيث يعاني الأطفال عادة من الأنيميا المصحوبة بضعف النمو العقلي والمعرفي. كانت حالات فيروس نقص المناعة البشرية تعد أبرز عوامل الخطورة لداء الاسطوانيات وعلى الرغم من أن عوامل الخطر الأخرى قد تم تحديدها للديدان الطفيلية لكن لم يكن أي منها ذات دلالة إحصائية لوجود الديدان الاسطوانية على وجه التحديد. كشفت العديد من الدراسات عن الديدان الخيطية في الكلاب وذباب الدروز غير القارص. ومع ذلك فهناك حاجة للمزيد من الدراسات المستقبلية للكشف عن دور هذه الخازنات في نقل الأمراض.

الخلاصة: تظهر هذه المراجعة أن الداء الخيطي مرض متغاضى عنه ومهمل في إثيوبيا. وهناك حاجة إلى منهج منظم يستخدم مزيج من الطرق التشخيصية الجزيئية والمصلية القائمة علي التأكد من الإصابة الحقيقية والعبء الواقع علي الداء الخيطي في اثيوبيا. وهناك حاجة أيضا للمزيد من الأبحاث لكسر دائرة نقل الأمراض بتحديد الخزانات البيئية، وعوامل الخطورة والكشف عن احتمالية الانتقال الحيواني.

Translated from English version into Arabic by Mona Eltantawy, proofread by Lara Khatro, through

TRANSLATORS
WITHOUT BORDERS

埃塞俄比亚类圆线虫病的风险因素、诊断、患病率和临床治疗效果的系统评价

Yitagele Terefe，Kirstin Ross 和 Harriet Whiley

摘要

引言：类圆线虫病是粪类圆线虫（*Strongyloides stercoralis*）引起的胃肠道感染。据估计，全球有3.7 亿人感染，主要分布在社会经济落后的热带和亚热带地区。

主要内容：本文对发表于过去十年的关于埃塞俄比亚类圆线虫病的危险因素、诊断、流行以及临床治疗效果的相关研究论文进行了分析。类圆线虫病在成年人中的流行率为 0.2%至 11.1%，儿童为 0.3%至 20.7%，艾滋病毒阳性成人为 1.5%至 17.3%，艾滋病毒阳性儿童为 5%。上述研究中主要使用显微镜镜检技术，该技术与血清学和 PCR 相比，可能导致患病率被低估 4 倍。在埃塞俄比亚，儿童的类圆线虫病是一个重要问题，因为患儿常患有贫血，可能导致精神和认知发育受损。类圆线虫病最重要的风险因素是 HIV 状态。虽然其他风险因素也被确认与蠕虫感染相关，但其相关性无统计学意义。目前在狗和不叮咬的环裂亚目蝇中检测到粪类圆线虫。然而，这些宿主在疾病传播的作用还需做进一步研究。

结论：该综述表明，在埃塞俄比亚，类圆线虫病是一种被忽视的疾病。需要一种基于分子和血清

学的综合诊断方法来确定埃塞俄比亚的类圆线虫病的真实发病率和疾病负担。还需要通过进一步研究来确定保虫宿主、风险因素和探索人兽共患转移的可能性，用于阻断该疾病的传播途径。

Translated from English version into Chinese by Cong-Shan Liu, edited by Pin Yang

CHINESE CENTER FOR DISEASE CONTROL AND PREVENTION
NATIONAL INSTITUTE OF PARASITIC DISEASES

La strongyloïdose en Éthiopie: revue systématique des facteurs de risque, du diagnostic, de la prévalence et des résultats cliniques

Yitagele Terefe, Kirstin Ross et Harriet Whiley

Résumé

Contexte: La strongyloïdose est une parasitose gastro-intestinale causée par le nématode *Strongyloides stercoralis*.On estime qu’elle affecte jusqu’à 370 millions de personnes dans le monde, principalement dans les régions tropicales et subtropicales économiquement défavorisées.

Discussion: Cette revue systématique de la littérature a identifié des études publiées au cours des dix dernières années, à propos des facteurs de risque, du diagnostic, de la prévalence et/ou des résultats cliniques de la strongyloïdose en Éthiopie. La prévalence de *S. stercoralis* variait entre 0,2 % et 11,1 % chez les adultes, 0,3 % et 20,7 % chez les enfants, 1,5 % et 17,3 % chez les adultes séropositifs au VIH ; elle était de 5 % chez les enfants séropositifs. Les études identifiées utilisaient principalement des méthodes par microscopie, qui ont potentiellement sous-estimé la prévalence d’un facteur quatre par rapport à la sérologie et à la RCP. La strongyloïdose infantile pose un problème particulièrement important en Éthiopie car les enfants sont souvent anémiés et cette anémie est associée à un retard du développement mental et cognitif. Le principal facteur de risque de strongyloïdose était le statut VIH. Bien que d’autres facteurs de risque aient été identifiés pour les helminthiases, aucun n’était statistiquement significatif dans le cas spécifique de *S. stercoralis*. Plusieurs études ont décelé *S. stercoralis* chez des chiens et chez des mouches non piqueuses du genre *Cyclorrhapha*. Les recherches futures devront explorer le rôle de ces réservoirs dans la transmission de la maladie.

Conclusions: Cette revue démontre que la strongyloïdose est une maladie méconnue et négligée en Éthiopie. Une approche systématique est nécessaire, en combinant des méthodes de diagnostic moléculaires et sérologiques afin d’en déterminer l’incidence et le fardeau réels dans ce pays. Des recherches supplémentaires sont également nécessaires pour rompre le cycle de la transmission en identifiant les réservoirs environnementaux, les facteurs de risque et en explorant le potentiel de transfert zoonotique.

Translated from English version into French by Suzanne Assenat, proofread by Iris Soliman, through

TRANSLATORS
WITHOUT BORDERS

Стронгилоидоз в Эфиопии: систематический обзор факторов риска, диагностики, уровня распространения и исходов болезни

Йитагале Терефе, Кирстин Росс и Харриет Уили

Аннотация

Предпосылки: Стронгилоидоз - инфекция желудочно-кишечного тракта, вызываемая гельминтом нематодой *Strongyloides stercoralis*. По оценкам, во всем мире стронгилоидозом инфицировано до 370 млн человек и он распространен он преимущественно в тропических и субтропических регионах с неблагоприятными социально-экономическими условиями.

Основной текст: В данном систематическом обзоре литературы были выделены исследования, опубликованные за последние десять лет, по факторам риска, диагностике, распространенности и/или исходу заболевания стронгилоидозом в Эфиопии. Распространенность *S. stercoralis* варьировала в пределах от 0,2 до 11,1% у взрослых, от 0,3% до 20,7% у детей, от 1,5% до 17.3% у ВИЧ-позитивных взрослых и до 5% у ВИЧ-позитивных детей. В рассматриваемых исследованиях главным образом использовались методы, основанные на микроскопии, при которых распространенность заболевания потенциально занижалась в четыре раза по сравнению с ПЦР или серологическими методами. Стронгилоидоз у детей представляет проблему чрезвычайной важности в Эфиопии, поскольку дети часто страдают анемией, сопровождающейся замедлением умственного и когнитивного развития. Наиболее значимым фактором риска заражения стронгилоидозом был ВИЧ-статус и, хотя для гельминтоза были выявлены и другие факторы риска, ни один из них не являлся статистически значимым специфично для *S. stercoralis*. В ходе некоторых исследований *S. stercoralis* были обнаружены у собак и некусающих циклорафанных мух. Однако, для изучения роли этих резервуаров передачи инфекции требуются дальнейшие исследования.

Заключение: Данный обзор показал, что стронгилоидоз в Эфиопии является упущенной и забытой болезнью. Для достоверного определения истинной заболеваемости и нагрузки на здравоохранение от стронгилоидоза в Эфиопии необходим системный подход с использованием комбинации молекулярных и серологических методов диагностики. Чтобы прервать цикл передачи стронгилоидоза требуются также дальнейшие исследования по определению резервуаров во внешней среде, факторов риска и изучение возможности передачи от животных к человеку.

Translated from English version into Russian by Nataliya Zhydkikh, proofread by Alexander Somin, through

TRANSLATORS WITHOUT BORDERS

Estrongiloidiasis en Etiopía: una revisión sistemática de los factores de riesgo, el diagnóstico, la prevalencia y la evolución clínica.

Yitagele Terefe, Kirstin Ross y Harriet Whiley

Resumen

Antecedentes: la estrongiloidiasis es una infección gastrointestinal causada por el nematodo parásito *Strongyloides stercoralis.* Se estima que infecta hasta a 370 millones de personas a nivel mundial y se encuentra sobre todo en zonas tropicales y subtropicales socioeconómicamente desfavorecidas.
Texto principal: la presente revisión bibliográfica sistemática identificó estudios publicados en la última década sobre los factores de riesgo, el diagnóstico, la prevalencia y la evolución clínica de la estrongiloidiasis en Etiopía. La prevalencia de *S. stercoralis* osciló entre el 0,2 % y el 11,1 % en adultos, el 0,3 % y el 20,7 % en niños, el 1,5 % y el 17,3 % en adultos VIH positivos y el 5 % en niños VIH positivos.Los estudios identificados utilizaron principalmente técnicas basadas en la microscopía que posiblemente subestimaron cuatro veces la prevalencia en comparación con la serología y la PCR. La estrongiloidiasis en los niños plantea un problema de particular importancia en Etiopía, ya que estos a menudo presentan anemia, la cual se relaciona con deficiencias en el desarrollo mental y cognitivo. El factor de riesgo más significativo para la estrongiloidiasis fue el estado de VIH y, a pesar de que se identificaron otros factores de riesgo para las infecciones por helmintos, ninguno fue estadísticamente significativo para *S. stercoralis* en particular. Varios estudios detectaron *S. stercoralis* en perros y moscas Cyclorrhapha no picadoras. Sin embargo, se necesitan investigaciones adicionales para examinar el papel de estos reservorios en la transmisión de la enfermedad.
Conclusiones: la presente revisión demostró que la estrongiloidiasis es una enfermedad olvidada y desatendida en Etiopía. Existe la necesidad de aplicar un enfoque sistemático que utilice una combinación de métodos de diagnóstico moleculares y serológicos a fin de determinar la incidencia y la carga verdaderas de la estrongiloidiasis en Etiopía. Asimismo, se necesita más investigación para romper el ciclo de transmisión mediante la identificación de los reservorios ambientales; los factores de riesgo, y el análisis del potencial zoonótico.

Translated from English version into Spanish by Mayra León, proofread by Maria Patricia, through

TRANSLATORS
WITHOUT BORDERS
